# Congenital Zika Syndrome in a Brazil-Paraguay-Bolivia border region: Clinical features of cases diagnosed between 2015 and 2018

**DOI:** 10.1371/journal.pone.0223408

**Published:** 2019-10-04

**Authors:** Fabio Antonio Venancio, Maria Eulina Quilião Bernal, Maria da Conceição de Barros Vieira Ramos, Neuma Rocha Chaves, Marcos Vinicius Hendges, Mattheus Marques Rodrigues de Souza, Márcio José de Medeiros, Cláudia Du Bocage Santos Pinto, Everton Falcão de Oliveira

**Affiliations:** 1 Programa de Pós-Graduação em Doenças Infecciosas e Parasitárias, Universidade Federal de Mato Grosso do Sul, Campo Grande, Brasil; 2 Centro Especializado em Reabilitação, Associação de Pais e Amigos dos Excepcionais, Campo Grande, Brasil; 3 Coordenadoria de Vigilância Epidemiológica, Secretaria Municipal de Saúde Pública de Campo Grande, Campo Grande, Brasil; 4 Faculdade de Medicina, Universidade Federal de Mato Grosso do Sul, Campo Grande, Brasil; 5 Universidade Federal do Rio de Janeiro, Campus Macaé, Rio de Janeiro, Brasil; 6 Instituto Integrado de Saúde, Universidade Federal de Mato Grosso do Sul, Campo Grande, Brasil; University of Oslo, NORWAY

## Abstract

Congenital Zika Syndrome (CZS) is a unique pattern of congenital abnormalities found in fetuses and neonates infected with the Zika virus (ZIKV). Here, we clinically identify and characterize infants with CZS between 2015 and 2018 in Mato Grosso do Sul, Brazil—a border area with Paraguay and Bolivia. This cross-sectional study, based on primary and secondary data, tracks the cases registered in the Brazilian Public Health Reporting System through the following stages: (1) preliminary data analysis, (2) identification of the congenital syndrome cases, (3) etiologic classification of the cases, (4) active search, and (5) clinical assessment. Of the 72 investigated cases, 16 were probable cases of CZS. Of these, it was only possible to clinically assess 11 infants. Considering the 16 probable cases of CZS, nine were classified as confirmed cases, and five as potential cases of the syndrome. Regarding clinical features, brain palsy was identified in all analyzed infants. Moreover, microcephaly and pseudobulbar syndrome were found in eight infants, and hydrocephalus was found in three individuals. In addition to these conditions, seven children were malnourished. Our study may provide significant insights for other researches that aim to elucidate CZS and its clinical and populational consequences.

## Introduction

Zika virus (ZIKV) is an arthropod-borne flavivirus from the *Flaviviridae* family—the same group of other arboviruses with epidemiological relevance, namely dengue, yellow fever, and West Nile viruses [1]. In Brazil, ZIKV was first isolated in April 2015, and 239,742 cases of ZIKV infection were reported between 2015 and 2018 [2,3]. From 2015 and 2016, there was an accelerated spread of ZIKV cases across the country along with an observed increase of microcephaly and other central nervous system abnormalities in newborn babies. These occurrences suggested that maternal ZIKV infection and congenital malformations could be linked [4–5].

Adult ZIKV infection is asymptomatic for 75–80% of the individuals. In symptomatic cases, however, the disease is often limited to unspecific manifestations that can last between three to 12 days [6]. Clinical manifestations of greater severity and consequence have also been described, such as that of congenital Zika syndrome (CZS). CZS is characterized by congenital anomalies in both fetuses and newborns infected with ZIKV during pregnancy [7]. This syndrome results from direct neurological damage caused by ZIKV replication in the neural progenitor cells and by promoting apoptosis or necrosis of neural cells. These cell-death events reduce the head volume and promote cortical calcification as well as ventricular enlargement [4,8].

The most common and recently reported pathologies in infants with CZS are microcephaly, central nervous system calcification, hydrocephalus, craniofacial disproportion; ocular, muscle tone, and postural alterations; exaggerated primitive reflexes, hyperexcitability, hyperirritability, arthrogryposis, and articular deformities [7,9,10].

Under the accelerated spread of ZIKV in Brazil, the Ministry of Health declared a Nationwide Public Health Emergency (ESPIN, in the Brazilian abbreviation) in November 2015 [11]. Later, the Ministry established a public health record to notify and investigate suspected cases of microcephaly caused by ZIKV infection during pregnancy. This system was termed RESP*-Microcephalia* [12] (*Registros de Eventos em Saúde Pública* and referred to as RESP henceforth). Since November 12^th^, 2016, cases of congenital anomalies due to syphilis, toxoplasmosis, rubella, cytomegalovirus, and herpes (STORCH) were also reported [9]. Importantly, before the RESP implementation, congenital anomalies diagnosed at birth were exclusively reported through a live-born declaration form via the Live-born Information System (*Sistema de Informações sobre Nascidos Vivos*–SINASC, in the Brazilian abbreviation).

Brazil’s Ministry of Health recommends that professionals perform a 2-year follow-up of newborn babies from mothers with confirmed ZIKV infections during pregnancy [12]. Given the clinical and epidemiological complexity of CZS and the short period since the ZIKV epidemy, it is crucial to characterize those affected to understand the relationship between the clinical features of affected children and congenital ZIKV infection. Thus, this study aimed to estimate the incidence and prevalence of CZS, as well as the incidence of ZIKV fever. In addition, our objective was to clinically profile the confirmed and potential cases of CZS in the Brazil-Paraguay-Bolivia border region.

## Material and methods

We performed a cross-sectional descriptive study on the magnitude of the ZIKV epidemic and CZS incidence in the state of Mato Grosso do Sul, Brazil. We considered all reported cases of both ZIKV fever and CZS from January 2015 through December 2018. This included all suspected and confirmed cases of congenital anomalies (CA) associated with the ZIKV infection.

The state of Mato Grosso do Sul is located in the Midwest Brazil, on the Brazil-Paraguay-Bolivia border region. The state has an 8,092,951 km^2^ area politically divided into 79 municipalities and has an estimated population of 2,748,023 individuals, with 2,097,238 of these residing in urban areas. The demographic density is 6.86 inhabitant/km^2^ [13].

### Data sources

Firstly, we assessed the following secondary data: (1) case reports of microcephaly and other CA as reported by the RESP and SINASC records, regardless of the etiology, and (2) ZIKV-exposed children who attended the *Centro Especializado em Reabilitação da Associação de Pais e Amigos dos Excepcionais* (CER-APAE) in Campo Grande, capital of Mato Grosso do Sul. CER-APAE is a state reference center that treats individuals with CA or other disabilities.

In order to quantify the burden of the ZIKV epidemic and its outcomes in Mato Grosso do Sul—especially in pregnant women—we also evaluated the ZIKV fever cases reported by the National System of Disease Notification (SINAN in the Brazilian abbreviation).

### Evaluation and tracking of reported congenital anomaly cases

Since RESP and SINASC may contain reports of CA due to other etiologies, and as there was no International Statistical Classification of Diseases and Related Health Problems (ICD-10) for CZS, we developed the following algorithm to track and identify CZS cases:

1. perform a preliminary analysis of all reported cases based on secondary data found in the RESP and SINAN databases,
2. identify CA cases (regardless of their etiology) and exclude the cases that did not fit any criteria of CA,
3. classify the CA cases based on their etiology through the analysis of diagnostic results of both the mother, the child, and from medical reports signed by expert physicians (infectious disease physician, neurologist, pediatrician, geneticist, and immunologist);
4. search for possible CZS cases, and
5. undertake a clinical assessment by a neuropediatrician to define and conclude the status of the cases.

**Fig 1:**
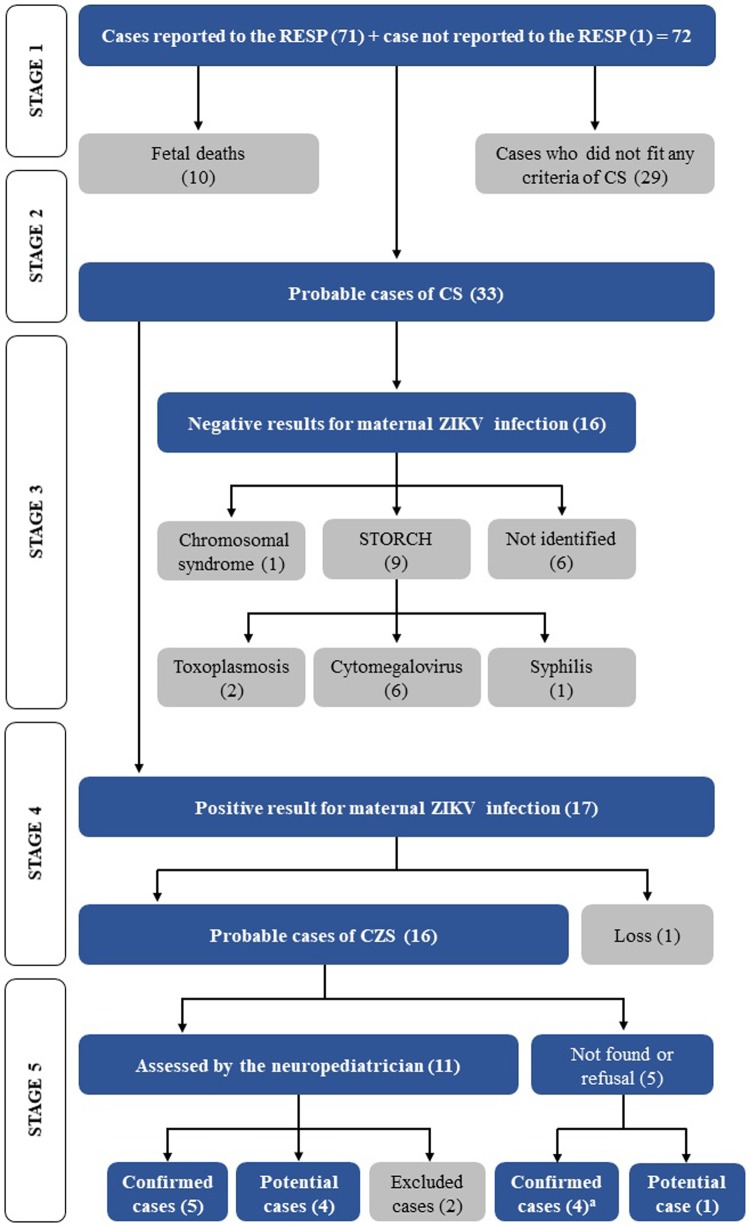
Algorithm for the screening of cases reported to the RESP^(a)^. (a)Inclusion of one case not reported to RESP, CA = congenital anomalies, CZS = congenital ZIKV syndrome. Note: Gray boxes indicate excluded cases during screening.

Fig 1 depicts the aforementioned algorithm used to identify the confirmed and unconfirmed cases of CZS.

Live-born infants reported to the RESP who did not fit the congenital anomaly criteria from the epidemiological surveillance guideline of the Brazilian Ministry of Health [9] were excluded from our initial analysis. These infants were not analyzed even when the mother had a confirmed ZIKV infection during pregnancy. For this, we defined CA as any defects in the constitution of any organ, or group of organs that causes abnormalities either structurally, functionally or morphologically, either present or not at birth, and caused by genetic, environmental, or mixed factors.

The status of maternal ZIKV-infections was analyzed using data from SINAN, and the Public Health Central Laboratory of Mato Grosso do Sul (LACEN-MS in the Brazilian abbreviation). Fetal deaths, reports with incomplete data, and cases that did not meet the criteria of CA were excluded from the study. The remaining cases were assessed and classified for their CZS status.

### CZS status classification and infant clinical assessment

To classify the CZS status, we used the following definitions:

1. probable case: a live-born child (reported to the RESP) from a ZIKV-positive mother (RT-PCR or ZIKV-reagent serology—IgM) reported in SINAN (classification based only on secondary data);
2. potential case: a live-born child with clinical outcomes and imaging evidence suggestive of CZS, with ZIKV-reagent serology (IgG) after birth, and/or live-born from a ZIKV-positive mother (RT-PCR or ZIKV-reagent serology—IgM), who has clinical outcomes and suggestive imaging evidence of CZS;
3. confirmed case: a live-born child with clinical outcomes and imaging evidence suggestive of CZS, RT-PCR ZIVK-positive or ZIKV-reagent serology (IgM) tested after birth and with unreacted/negative STORCH results in both the mother and newborn.

The following criteria were used to classify the cases of CA caused by other etiologies:

1. live-born babies with CA who had a negative laboratory result for ZIKV infection for both mother and child, in addition to a positive outcome for at least one of the following: syphilis, toxoplasmosis, rubella, cytomegalovirus, or herpes simplex (STORCH);
2. live-born babies presenting CA caused by chromosomal alterations, or through other non-infectious causes.

A neuropediatrician conducted a clinical assessment to characterize the clinical status at the time of the study. This assessment—based on recommendations from Brazil’s Health Ministry [14]—focused on both the cognitive and motor development of the children. Additional clinical information was also gathered through interviews with the babies’ guardians, and through data available from the Brazilian Child Health Register.

In unlocated or unassessed cases, the infection status of the mother and newborn infant was inferred using data from the Health Surveillance Strategical Information Center (*Centro de Informações Estratégicas em Vigilância em Saúde*–CIEVS in the Brazilian abbreviation) of Mato Grosso do Sul.

### Data analysis

A descriptive analysis and characterization of the cases were conducted–this comprised of determining the frequency distribution of the chosen variables, determining the mean, and performing an analysis of proportions. A crude incidence of ZIKV fever, CZS, and cumulative prevalence of CZS was also obtained.

An estimate of the incidence of ZIKV fever in the total population was first found by calculating the ratio between the number of such cases reported in SINAN by the total state population. For the incidence of ZIKV fever in pregnant women, we used both the RT-PCR ZIVK-positive and ZIKV-reagent serology-positive data for pregnant women as reported in SINAN, along with the total number of births according to data from SINASC. To estimate the incidence of CZS, we used the number of confirmed cases after the screening and the total number of births from SINASC. This data is shown in the S1 and S2 Tables.

For anonymity, a case identification number was given to all children being investigated in the study. This number was used during all stages of our analysis.

## Ethics

This study was approved by the Research Ethics Committee of the Federal University of Mato Grosso do Sul (CAAE: 91326518.1.0000.0021) and registered under number 3.298.330. All individuals included and clinically assessed in this study signed the participant consent form. For the minors, parents or guardians of the participating children signed consent forms on the same day that the participants entered the study.

## Results

### CZS and ZIVK fever cases

**Fig 2:**
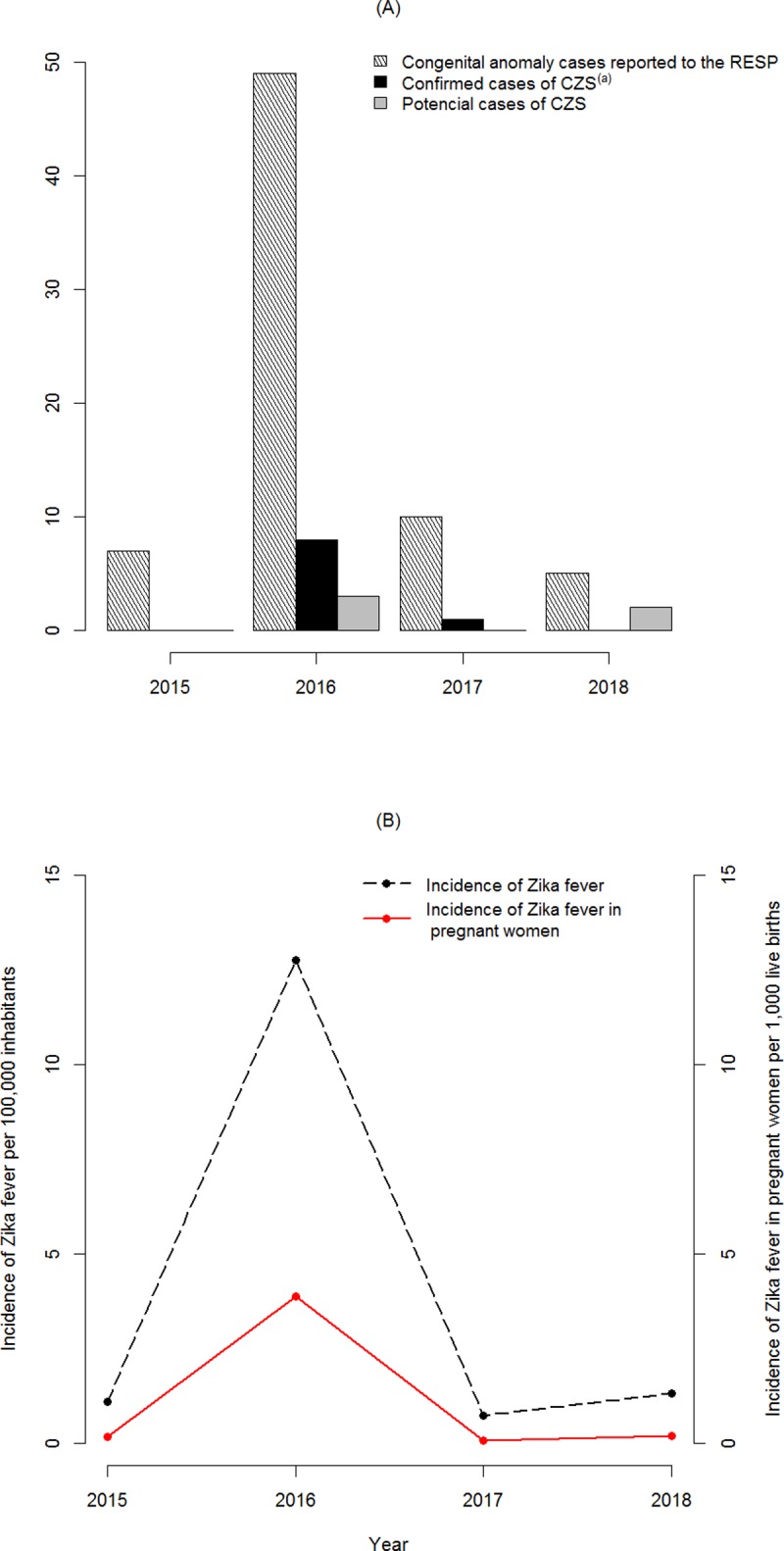
**Frequency of congenital anomaly cases reported to the RESP, confirmed and potential cases of CZS (A), and ZIKV fever incidence in total population and pregnant women (B).** (a) It includes four unassessed cases that were classified as confirmed based on secondary data.

In Mato Grosso do Sul, from January 2015 through December 2018, 71 cases were reported to the RESP (Fig 2A) with either suspicion or diagnosis of congenital anomalies. Most occurrences were from Campo Grande (24 cases), while the other 47 cases were distributed over 27 municipalities. During the same period, 1,082 cases of CA were reported in SINASC, of which 37 cases were of microcephaly (S1 Table). Of the 37 reports found in SINASC, only nine were reported to the RESP. Importantly, the RESP included only cases of congenital anomalies related to STORCH and ZIKV. Therefore, we carried out the analysis and screening of CZS cases based on the RESP. The data available in SINASC was used only to supplement the necessary information, such as clinical data from children at delivery.

In 2015, seven cases were reported; however, all of these were excluded from our analysis as they did not fit the definition for CA or CZS previously described. In 2016, 49 cases were reported—three of which were classified as potential cases and eight as confirmed. In the next two years, the number of reports decreased, with 10 in 2017 (one being confirmed), and five reports in 2018, with two being potential CZS cases. Therefore, the cumulative prevalence of CZS was 0.20 cases per 1,000 live-born babies. The S1 Table shows this data by year.

Regarding ZIKV fever in the total population, 427 cases of ZIKV infection were confirmed through laboratory analyses (a crude cumulative incidence of 15.83 cases per 100,000 inhabitants) (Fig 2B). Among these 427 cases, 185 were pregnant women, which resulted in a cumulative incidence of 4.21 cases per 1,000 live-born babies. In 2016, 166 pregnant women were RT-PCR ZIVK-positive or ZIKV-reagent serology-positive (Fig 2B). This represents 89.7% of the total pregnant women reported with ZIKV infection from 2015 to 2018. The data is shown by year in the S2 Table.

### Evaluation and tracking of reported congenital anomaly cases

Of the total number of reports found in RESP, ten fetal death cases occurred among the 71 cases registered. Considering only fetal deaths, four cases were ZIKV-positive, two were classified as likely infected because only the mother had a confirmed ZIKV infection, and one case was inconclusive for CZS. These classifications were made by the city’s epidemiological surveillance service. Of the 61 remaining cases, 29 were excluded because they did not fit with the congenital criteria.

The criteria used to determine the presence of microcephaly was based on the WHO metrics [15] and governmental criteria of head circumference measurements smaller than 2 standard deviations (or Z-scores) below the mean (Table 1). Microcephaly was reported in the RESP for 29 cases, for which only ten were diagnosed during the antenatal period. We found one child in the CER-APAE with substantial clinical and laboratory CZS who was not reported to the RESP (case 10 in Table 1). With a culmination of 33 cases, the third step of the tracking algorithm was initiated (Fig 1 and Table 1).

**Table 1:** Clinical and laboratory data from mother and children from the RESP by location.

ID	City	Year of birth (child)	Initial clinical findings	Period of identification of the clinical finding	Confirmation of laboratory infection for ZIKV in the infant (method)	Confirmation of laboratory infection for ZIKV in the mother (method)	Etiology of congenital anomaly reported to the RESP
1	Campo Grande	2016	Microcephaly	Postpartum	Yes (RT-PCR)	Yes (RT-PCR)	ZIKV
2	Campo Grande	2016	Microcephaly	Postpartum	Yes (RT-PCR)	Yes (RT-PCR)	ZIKV
3	Caracol	2016	Microcephaly	Intrauterine	Yes (RT-PCR)	Yes (RT-PCR)	ZIKV
4	Campo Grande	2016	Microcephaly	Intrauterine	Yes (RT-PCR)	Yes (RT-PCR)	ZIKV
5	Campo Grande	2016	Microcephaly	Intrauterine	Yes (RT-PCR)	Yes (RT-PCR)	ZIKV
6	Campo Grande	2016	Microcephaly	Postpartum	Yes (RT-PCR)	Yes (RT-PCR)	ZIKV
7	Camapuã	2017	Microcephaly	Postpartum	Yes (RT-PCR)	Yes (RT-PCR)	ZIKV
8	Dourados	2016	Microcephaly	Postpartum	Yes (RT-PCR)	Yes (RT-PCR)	ZIKV
9	Campo Grande	2016	Microcephaly	Intrauterine	Yes (RT-PCR)	Yes (RT-PCR)	ZIKV
10^**(a)**^	Campo Grande	2016	Not registered	____	No	Yes (RT-PCR)	ZIKV
11	Campo Grande	2018	Microcephaly	Intrauterine	Yes (Serology anti-ZIKV—IgG)	Yes (Serology anti-ZIKV—IgG)	ZIKV
12	Campo Grande	2016	Brain Calcifications	Intrauterine	No (RT-PCR)	Yes (RT-PCR)	No data available
13	Rio Verde	2016	Microcephaly	Intrauterine	Yes (Serology anti-ZIKV—IgG)	Yes (RT-PCR)	ZIKV
14	Campo Grande	2018	Microcephaly	Postpartum	Yes (Serology anti-ZIKV—IgG)	Yes (Serology anti-ZIKV—IgG)	ZIKV
15	Caarapó	2016	Syndactilism, Polydactylism and Palatine Fissure	Postpartum	No (RT-PCR)	Yes (RT-PCR)	No data available
16	Campo Grande	2016	Microcephaly	Postpartum	No (RT-PCR)	Yes (RT-PCR)	No data available
17	Bonito	2016	Microcephaly	Intrauterine	No (RT-PCR)	No (RT-PCR)	Cytomegalovirus
18	Campo Grande	2016	Microcephaly	Postpartum	No (RT-PCR)	No (RT-PCR)	Cytomegalovirus
19	Dourados	2016	Microcephaly	Postpartum	No (RT-PCR)	No (RT-PCR)	*Toxoplasma gondii*
20	Dourados	2016	Microcephaly	Postpartum	No (RT-PCR)	No (RT-PCR)	Not identified
21	Fátima do Sul	2016	Microcephaly	Postpartum	No (RT-PCR)	No (RT-PCR)	Cytomegalovirus
22	Jardim	2017	Microcephaly	Postpartum	No (RT-PCR)	No (RT-PCR)	No data available
23	Nova alvorada do Sul	2018	Microcephaly	Postpartum	No (RT-PCR)	No (RT-PCR)	Not identified
24	Nova Andradina	2016	Microcephaly	Intrauterine	No (RT-PCR)	No (RT-PCR)	Not identified
25	Paranaíba	2016	Microcephaly	Postpartum	No (RT-PCR)	No (RT-PCR)	*Toxoplasma gondii*
26	Paranaíba	2016	Brain Calcifications	Intrauterine	No (RT-PCR)	No (RT-PCR)	No data available
27	Ponta Porã	2015	Microcephaly	Postpartum	No (RT-PCR)	No (RT-PCR)	Cytomegalovirus
28	Ponta Porã	2017	Microcephaly	Postpartum	No (RT-PCR)	No (RT-PCR)	Cytomegalovirus
29	Ponta Porã	2016	Microcephaly	Postpartum	No (RT-PCR)	No (RT-PCR)	*Treponema Pallidum*
30	Paranaíba	2015	Microcephaly	Postpartum	No (RT-PCR)	Yes (RT-PCR)	Not identified
31	Maracaju	2016	Microcephaly	Postpartum	No (RT-PCR)	No (RT-PCR)	Cytomegalovirus
32	Campo Grande	2018	Microcephaly	Postpartum	No (RT-PCR)	No (RT-PCR)	Genetic mutation
33	Campo Grande	2016	Microcephaly	Postpartum	No (RT-PCR)	No (RT-PCR)	Not identified

^(a)^ Child not reported to the RESP; ID = identification number; RT-PCR = reverse transcription polymerase chain reaction.

At this point, 16 mothers had negative laboratory outcomes for ZIVK infection, for which we concluded that nine cases were related to STORCH (cases 17, 18, 19, 21, 25, 27, 28, 29, and 31 shown Table 1), one case with a chromosomal mutation (case 15 in Table 1), and an additional six cases where it was not possible to identify the etiology of the congenital anomaly diagnosed in the infant (cases 20, 23, 24, 30, 32, 33 in Table 1). Regarding the nine cases related to STORCH, six had a cytomegalovirus positive diagnosis (reactive IgM and IgG antibodies in the mother and reactive IgM in the child), two cases showed positive serology for toxoplasmosis (reactive IgM antibody in the mother and the infant), and one had confirmation for congenital syphilis. These 16 cases were discarded for CZS (Table 1 and Fig 2).

In the fourth step of the screening algorithm, of the 33 eligible children, there were 17 cases whose mothers presented a positive laboratory analysis (RT-PCR and/or serology) for the ZIKV infection. However, at this point, we were unable to obtain data for one child, which consequently meant that this individual had to be excluded from the study. With this, only 16 infants who were initially classified as probable cases of CZS based on secondary data were investigated. Of these, 13 mother-infant pairs had a positive laboratory result for ZKIV infection in addition to registered clinical data fitting a diagnosis of CZS. Also, three infants had negative laboratory results but positive clinical data for CZS (with mothers infected with ZIKV).

From the 16 probable cases, two were removed from our analyses: one family moved to a different state in Brazil, and the other was an indigenous infant who could not be located. Furthermore, parents and legal guardians of three children did not authorize their participation in our study. Based on secondary data (clinical reports found on RESP and laboratory outcomes for the mother-infant pairs), our study classified four infants as being confirmed CZS cases (cases 6, 7, 8, 9 in Tables 1 and 2) and one as a CZS potential case. Therefore, for the last step of the screening algorithm, only 11 infants were clinically assessed by the neuropediatrician.

**Table 2:** Clinical characteristics and laboratory data of probable cases of CZS at delivery (before clinical assessment).

ID	Sex	Pregnancy period (in week)	Laboratory confirmation for infant	Method of laboratory evidence	Maternal infection	Trimester of maternal infection	Previous maternal infection by other arbovirus	Period of malformation identification	HC at delivery	SD
1	M	39	Yes	RT-PCR	Yes	Second	Yes^(a)^	Postpartum	29	-3
2	M	42	Yes	RT-PCR	Yes	First	Yes^(a)^	Postpartum	31	-3
3	F	39	Yes	RT-PCR	Yes	First	No	Antenatal (32w)	28	-4
4	M	35	Yes	RT-PCR	Yes	First	No	Antenatal (35w)	28	-3
5	F	36	Yes	RT-PCR	Yes	Third	No	Antenatal (33w)	26	-4
6	M	34	Yes	RT-PCR	Yes	Second	No	Postpartum	31	0
7	M	39	Yes	RT-PCR	Yes	—-	No	Postpartum	32	-1
8	F	37	Yes	RT-PCR	Yes	—-	No	Postpartum	29	-2
9	M	39	Yes	RT-PCR	Yes	First	No	Antenatal (30w)	31	-2
10	M	41	No	RT-PCR	Yes	First	No	Postpartum	35,5	0
11	F	34	Yes	Serology (anti-ZIKV—IgG)	Yes	Unknown	No	Antenatal (32w)	26	-4
12	M	37	No	RT-PCR	Yes	Second	Yes^(a)^	Antenatal (36w)	32	-1
13	M	39	Yes	Serology (anti-ZIKV—IgG)	Yes	First	No	Antenatal (13w)	30	-3
14	F	35	Yes	Serology (anti-ZIKV—IgG)	Yes	—-	No	Postpartum	29	-2
15	F	37	No	RT-PCR	Yes	First	No	Postpartum	32,5	0
16	M	32	No	RT-PCR	Yes	Second	No	Postpartum	29	0

^(a)^ Previous historical to dengue; ID = identification number; SD = standard deviation; HC = head circumference; RT-PCR = Reverse Transcription Polymerase Chain Reaction; —- = no information available

Following the clinical assessments, five cases were found to be confirmed diagnoses of CZS while another four were defined as being potential CZS cases. The remaining two were discarded as one did not fit any criteria of CA, and the other individual was classified as having CA resulting from chromosomal alterations.

### Clinical outcomes of probable CZS cases at birth (before clinical assessment)

Table 2 shows the clinical outcomes of 16 infants at birth who were initially classified as probable cases of CZS at the beginning of step 4. Among those, 12 cases had their ZIKV exposure confirmed (nine by RT-PCR and three by serology for IgG anti-ZIKV). The three children positive for IgG anti-ZIKV were classified as potentially infected with ZIKV during pregnancy.

There was no laboratory evidence for ZIKV infection for the other four children, and based on their clinical criteria and maternal status (RT-PCR ZIVK-positive), they remained as probable cases (Table 2). At this point, we highlight case 12, who presented with a head circumference under the average standard deviation (32 cm) at delivery, which then evolved to microcephaly some weeks after birth.

Regarding the period of infection, 11 women were infected with ZIKV in the first or second trimester of pregnancy. The mother of case 11 was asymptomatic during pregnancy, and so we were unable to determine when maternal infection occurred. Additionally, three women self-reported dengue before pregnancy.

### Clinical outcomes of confirmed and potential cases of CZS (during clinical assessment)

Table 3 presents the clinical features of the 11 children assessed by a neuropediatrician. Of these, two cases were CZS negative: one was excluded because of a chromosomal mutation (case 15), and the other was discarded for CA (case 16). Therefore, the following results include nine cases that fit the criteria of either confirmed or potential CZS. The minimum age was seven months, and the maximum was two years and four months, with a mean age of 2 years and one month.

**Table 3:** Clinical outcomes of cases assessed in stage 5.

ID	Child age (at assessment)	Current HC	SD	CP	Most affected brain hemisphere	Microcephaly	Craniofacial disproportion	Redundant Scalp Skin	Hydrocephalus	Epilepsy	Arthrogryposis	Visual and oculomotricity alterations	Auditory Impairment	Nutritional Status	Final classification for CZS
1	2y 3m 29d	42	-4	Yes	Left	Yes	Yes	Yes	Yes	No	No	No	No	Malnutrition	Confirmed
2	2y 3m 20d	39	-4	Yes	Not identified	Yes	Yes	Yes	No	Yes	No	Yes	No	Malnutrition	Confirmed
3	2y 3m 7d	38	-4	Yes	Left	Yes	Yes	Yes	No	Yes	No	Yes	No	Malnutrition	Confirmed
4	2y 4m 3d	36,5	-4	Yes	Left	Yes	Yes	Yes	No	Yes	No	Yes	No	Malnutrition	Confirmed
5	2y 4m 14d	36	-4	Yes	Right	Yes	Yes	Yes	No	Yes	No	No	No	Malnutrition	Confirmed
10	2y 4m 14d	49	0	Yes	Not identified	No	No	No	No	No	No	No	No	Eutrophy	Potential
11	0y 7m 12d	36	-4	Yes	Right	Yes	Yes	Yes	No	No	No	No	No	Obesity	Potential
12	2y 3m 22d	42	-4	Yes	Left	Yes	Yes	Yes	Yes	Yes	No	Yes	Yes	Malnutrition	Potential
13	2y 3m 13d	42	-4	Yes	Right	Yes	Yes	Yes	Yes	Yes	No	No	No	Malnutrition	Potential
15	2y 6m 25d	45	-2	No	Not applicable	Yes	No	No	No	No	No	No	No	Eutrophy	Discarded
16	2y 6m 11d	49	0	No	Not applicable	No	No	No	No	No	No	No	No	Eutrophy	Discarded

ID = identification number; HC = head circumference; SD = standard deviation; CP = cerebral palsy.

Regarding developmental brain impairment, we observed that four infants had their left hemisphere affected to a greater extent, while three children showed more abnormalities in their right hemisphere. We were unable to identify the affected brain hemisphere for the remaining two cases (cases 2 and 10). Moreover, eight children were diagnosed with microcephaly, craniofacial disproportion, and redundant scalp skin. At the time of assessment, all children diagnosed with microcephaly had a head circumference smaller than 4 standard deviations (SD) below the mean. Among these cases, two mothers with confirmed ZIKV fever in their first and second trimester of pregnancy reported that they were informed about their children's diagnosis of microcephaly only after delivery. Lastly, brain palsy and motor impairment were detected in the nine children, with hydrocephalus being diagnosed in five of those cases, and eight presenting bilateral paresis. Four of these individuals with right-hemisphere abnormalities displayed a higher degree of impairment.

In observance of ocular outcomes, the neuropediatrician determined visual impairment and oculomotricity alterations in four cases. Cases 2, 4, and 12 revealed atrophy in the optical nerve as well as microlesions of the retina with a low standard of vision. Additionally, case 4 was also diagnosed with glaucoma and strabismus.

Arthrogryposis was not diagnosed in the children assessed in this study. Motor impairment was diagnosed in all children and bilateral paresis in eight children. The child without microcephaly (case 10) showed a less severe motor impairment and absence of bilateral paresis. In addition, eight infants had a confirmed diagnosis for pseudobulbar syndrome, and all presented pervasive developmental disorder. Uniquely, case 10 did not show such outcomes for pseudobulbar syndrome, with only dysphonia being diagnosed. Regarding auditive impairments, there was only one positive case, which was diagnosed with bilateral auditory loss (case 12). Lastly, seven individuals were malnourished with a < 3^rd^ percentile in the body-mass index curve for children, while case 11 was diagnosed with obesity (99^th^ percentile).

## Discussion

In contrast to other studies that quantified CZS in other regions [16–18], we employed primary data obtained from both an active search and by performing clinical assessments of infants; in addition to screening secondary data from RESP, SINASC and SINAN. Through this method, we determined the clinical status of infants with CZS after almost two years following the ZIKA epidemics in Mato Grosso do Sul. Our results showed that the clinical outcomes are highly similar between both the confirmed and potential CZS cases.

Similarly, in other studies [17–18], most CZS cases were diagnosed in 2016; however, new cases are still reported despite the end of the ZIKV fever epidemic [3]. The existence of one confirmed CZS case in 2017 and two more occurrences of potential CZS in 2018 shows that its cumulative prevalence has increased even with a decreased incidence of ZIKV fever in pregnant women and the general population (S1 and S2 Tables). This scenario reinforces the notion that CZS is still a public health problem and that pregnant women should still take precautions.

Brazil’s Ministry of Health confirmed that there are 3,332 cases of congenital syndrome related to ZIKV or other etiologies among the 17,041 cases reported in the RESP [19]. Through this investigation, 71 cases registered in the RESP for Mato Grosso do Sul had their status defined, also comprising of the discarded cases that were incorrectly classified as CZS. Of this, it was found that 32 infants had a positive diagnosis for congenital syndrome due to different etiologies.

Governmental data from the Ministry of Health of Brazil shows that the incidence of CZS varies regionally within the country, with the highest frequency being found to the Northeast, comprising 58.5% of all reported cases [19]. In contrast, between 2015 to 2017, it is seen that the total number of pregnant women suffering from ZIKV fever was lower in the Northeast (with 6,042 cases) when compared to the Southeastern region (9,023 cases). In that same period, a total of 2,908 pregnant women with ZIKV fever were reported in the Midwest region [20].

The crude cumulative incidence of ZIKV fever in pregnant women in the state of Rio de Janeiro (11.88 cases per 1,000 live-born babies), located in the Southeast of Brazil, was almost three times higher compared to Mato Grosso do Sul between 2015 to 2018. However, when we compared the cumulative incidences of CZS for the same period, the results were very similar, with an incidence of 0.20 cases per 1,000 live-born babies born for both states [21–22].

Regarding STORCH related cases among the total reports, data showed that cytomegalovirus *Toxoplasma gondii* and *Treponema pallidum* were identified as teratogenic etiologic agents for some cases. These agents were already present in other studies classifying RESP data [16–18]. Similarly to the CZS, these infectious etiologies also produce congenital syndromes leading to microcephaly [9,23,24]. Additionally, they result in other abnormalities also present in CZS cases, including brain calcifications [9,21], hydrocephalus, intrauterine growth retardation [9], and ocular lesions [9,24].

Only 7.5% (n = 14) of the infants born from women infected with ZIKV (n = 185) revealed clinical and laboratory evidence of CZS in Mato Grosso do Sul. At present, it is unclear as to which mechanisms are responsible for an increasing or decreasing the risk of fetuses developing of CZS during maternal ZIKV infection [25–28]. To date, it has been described that viral transmission through the placenta can occur at any time during pregnancy [29–31], but the transmission is not necessarily associated with the acute phase of the disease in pregnant women [32]. Although some studies reported evidence suggesting that prior dengue virus (DENV) infection might confer cross-immunity against ZIKV infection [33,34], both Zimerman et al. [35] and Brown et al. [36] have shown that, in maternal ZIKV infection, specific anti-DENV antibodies favor greater placental ZIKV viral replication. This may significantly increase the placental tissue damage and therefore result in the restriction of fetal growth.

Furthermore, Pedrosa et al. [37] described that saxitoxin, a neurotoxin produced by the freshwater cyanobacteria *Raphidiopsis raciborskii* found in South America, doubled the amount of ZIKV-induced neural cell death in progenitor areas of human brain organoids. It was also observed that the chronic ingestion of water contaminated with this toxin before and during pregnancy caused brain abnormalities in the offspring of ZIKV-infected immunocompetent mice. The authors’ data showed that saxitoxin-producing cyanobacteria is overspread in the water reservoirs of Northeast Brazil and may have acted as an adjuvant and potentiated the outcomes of ZIKV epidemics in Brazil.

Most of the ZIKV fever symptoms were reported during the first and second trimester of pregnancy, as shown elsewhere [16,17]. Recently, evidences indicate that if the maternal infection occurs before conception, until ten days later, or by the end of pregnancy, ZIKV would not offer a high risk for developing microcephaly [38]. Nevertheless, Brasil et al. [39] found central nerve system abnormalities in a newborn from a mother infected with ZIKV in the 39^th^ week of pregnancy. This observation is in keeping with our findings from one case, whose symptoms of ZIKV fever were reported by the mother in the third trimester of pregnancy and subsequently led to CZS development in the newborn (case 5).

Craniofacial disproportion and redundant scalp skin [10] (another differential mark of CZS microcephaly) were observed in all infants assessed with microcephaly. Those findings are in accordance with features already described elsewhere [4,39,40]. Microcephaly–commonly seen in the most severe cases of CZS–is related to the viral strain found in Brazil [41]. Phylogenetic analyses revealed that the Brazilian strain (ZIKV^BR^) comes from an Asian lineage [42]. However, the presence of microcephaly is related to the Brazilian viral lineage because, despite the Asian derivation, there were adaptive changes in human cells over 70 years [41].

Regarding the clinical aspects of our findings, the presence of hydrocephalus was observed in three of the 11 infants assessed by the neuropediatrician. This symptom was recently described as constitutive of the clinical definition for CZS [43]. The presence of epileptic seizures approximately three months after birth was also detected following our screening [40].

Pseudobulbar affect (PBA) is a condition characterized by dysphagia, dysarthria, and dysphonia; these are manifested by a deficit in the voluntary movements of the tongue and facial muscles, in addition to emotional lability. PBA may be related to the impairment of motor fibers between the cerebral cortex to the lower brain stem [44]. Leal et al. [45] observed orofacial abnormalities when assessing dysphagia in children presenting CZS. This suggests that PBA in CZS cases are not only related to damages in cortex and brain stem. Dysphagia could also be directly associated with both malnutrition and low body weight for children suffering brain palsy [46], as observed in our study.

Although our study did not aim to assess database quality and the agreement between SINAN and RESP datasets, some differences were found. The most notable was regarding SINAN, for which of six women whose children developed CZS were unreported. Moreover, we identified one case of CZS that was not reported to the RESP, indicating that there may be inconsistencies with the notification, investigation, and epidemiological surveillance systems. Importantly, we observed that the average time for the Ministry of Health to process and receive the information of newborn babies takes approximately 30 days [12]. This delay is problematic, because may postpone the beginning of procedures of early stimuli in affected children.

Another important point highlighted from the RESP data was the identification of microcephaly only after delivery in 19 of the 29 registered cases. By exclusively analyzing data from children with CZS, we observed that six of the 14 children had microcephaly identified only after delivery. While performing the clinical assessments, three mothers had reported the identification of microcephaly (with 3 SD below mean) only at birth, despite a diagnosis of ZIKV fever in their early pregnancy period and an adequate follow-up involving antenatal consultations. A similar situation was described in the state of Rio de Janeiro, where only 60% of microcephaly cases were identified in the postnatal period [22].

Some limitations of this research include: (1) five probable cases of CZS that could not be assessed, (2) the quality of data found in the governmental databases used—especially the RESP and SINAN—due to either the absence of records, or inconsistency between the two databases when maternal data were compared, and (3) underreporting, as evidenced by a CZS case which was not present in the RESP. Furthermore, no reports of microcephaly or other CA related to ZIKV-infection were found in SINAN. However, as previously mentioned, this fact could be due to the absence of ICD for these outcomes during the ZIKV epidemic. Our study provided significant data for the quantification of CZS cases in Mato Grosso do Sul, as well as the classification of congenital syndromes caused by other etiologies. Thus, we conclude that it is crucial to continually examine these infants, given the possibility of continuous viral replication of the ZIKV in the brain of children infected during pregnancy [47]. This is also important for cases where infants did not subsequently present congenital abnormalities.

Our findings may help support both societal needs, as well as public policy requirements focused on reducing the damages caused by the CZS. This is especially true when considering its magnitude in Brazil and Latin America. In addition, we suggest that the regional health service should implement new actions to reduce ZIKV infections and to manage strategies for identifying cases with the clinical aspects described.

## Supporting information

S1 Table Frequencies of live-born, congenital anomaly cases reported to SINASC and RESP, confirmed and potential cases of CZS, incidence and prevalence of CZS, according to year, Mato Grosso do Sul, Brazil, 2015 to 2018. SINASC = Brazilian Live-born Information System; RESP = *Registros de Eventos em Saúde Pública*; CZS = congenital ZIKV syndrome.
(PDF)

S2 Table Estimated total population, number of live-born, Zika fever cases reported to SINAN, and ZIKV fever incidence, according to year, Mato Grosso do Sul, Brazil, 2015 to 2018. SINAN = National System of Disease Notification of Brazil; RESP = *Registros de Eventos em Saúde Pública*.
(PDF)

S1 Text Glossary of abbreviations. (PDF)
