# Correction: Congenital Zika Syndrome in a Brazil-Paraguay-Bolivia border region: Clinical features of cases diagnosed between 2015 and 2018

**DOI:** 10.1371/journal.pone.0224842

**Published:** 2019-10-30

**Authors:** Fabio Antonio Venancio, Maria Eulina Quilião Bernal, Maria da Conceição de Barros Vieira Ramos, Neuma Rocha Chaves, Marcos Vinicius Hendges, Mattheus Marques Rodrigues de Souza, Márcio José de Medeiros, Cláudia Du Bocage Santos Pinto, Everton Falcão de Oliveira

The Funding statement is incorrect. The correct Funding statement is as follows: This study was financed in part by the Coordenação de Aperfeiçoamento de Pessoal de Nível Superior–Brasil (CAPES) (https://www.capes.gov.br/) and by Universidade Federal de Mato Grosso do Sul (UFMS) (https://www.ufms.br/). The funders did not have any additional role in the study design, data collection and analysis, decision to publish, or preparation of the manuscript.
