# Supplementary material for: Congenital Zika Syndrome in a Brazil-Paraguay-Bolivia border region: Clinical features of cases diagnosed between 2015 and 2018
Source: PLoS One. 2019 Oct 4;14(10):e0223408. doi: 10.1371/journal.pone.0223408 (PMC6777783; doi:10.1371/journal.pone.0223408)
Supplement: S1 Table — SINASC = Brazilian Live-born Information System; RESP = Registros de Eventos em Saúde Pública; CZS = congenital ZIKV syndrome. (PDF) [file pone.0223408.s001.pdf]

Supporting information

S1 Table. Frequencies of live-born, congenital anomalies reported to SINASC and RESP, confirmed and potential cases of CZS, incidence and prevalence of CZS, according to year, Mato Grosso do Sul, Brazil, 2015 to 2018.

Year	Live-born	Congenital anomalies reported to SINAN		Congenital anomalies reported to RESP	CZS confirmed cases	CZS potential cases	CZS crude incidence	CZS cumulative prevalence
		Total	Microcephaly					
**2015**	44,630	248	6	7	0	0	0.00	0.00
**2016**	42,822	282	24	49	8	3	0.19	0.19
**2017**	44,996	298	4	10	1	0	0.02	0.20
**2018**	44,261	254	3	5	0	2	0.00	0.20

SINASC = Brazilian Live-born Information System; RESP = *Registros de Eventos em Saúde Pública*; CZS = congenital Zika virus syndrome.
