# Supplementary material for: Congenital Zika Syndrome in a Brazil-Paraguay-Bolivia border region: Clinical features of cases diagnosed between 2015 and 2018
Source: PLoS One. 2019 Oct 4;14(10):e0223408. doi: 10.1371/journal.pone.0223408 (PMC6777783; doi:10.1371/journal.pone.0223408)
Supplement: S2 Table — SINAN = National System of Disease Notification of Brazil; RESP = Registros de Eventos em Saúde Pública. (PDF) [file pone.0223408.s002.pdf]

Supporting information

S2 Table. Estimated total population, number of live-born, Zika fever cases reported to SINAN, and ZIKV fever incidence, according to year, Mato Grosso do Sul, Brazil, 2015 to 2018.

Year	Estimated population	Live-born	Zika fever cases	Zika fever cases in pregnant women	Zika fever incidence	Zika fever incidence in pregnant women
**2015**	2,651,235	44,630	29	8	1.09	0.18
**2016**	2,682,386	42,822	342	166	12.75	3.88
**2017**	2,713,147	44,996	20	3	0.74	0.07
**2018**	2,748,023	44,261	36	8	1.31	0.18

SINAN = National System of Disease Notification of Brazil; RESP = *Registros de Eventos em Saúde Pública*.
