# Supplementary material for: Congenital Zika Syndrome in a Brazil-Paraguay-Bolivia border region: Clinical features of cases diagnosed between 2015 and 2018
Source: PLoS One. 2019 Oct 4;14(10):e0223408. doi: 10.1371/journal.pone.0223408 (PMC6777783; doi:10.1371/journal.pone.0223408)
Supplement: S1 Text — (PDF) [file pone.0223408.s003.pdf]

Supporting information

S1 Text. Glossary of abbreviations

CA	Congenital anomalies
CER-APAE	Centro Especializado em Reabilitação da Associação de Pais e Amigos dos Excepcionais
CIEVS	Health Surveillance Strategical Information Center
CP	Cerebral palsy
CZS	Congenital Zika Syndrome
DENV	Dengue virus
ESPIN	Nationwide Public Health Emergency
IgG	Immunoglobulin G
IgM	Immunoglobulin M
ICD	International Statistical Classification of Diseases and Related Health Problems
ID	Identification number
HC	Head circumference
LACEN-MS	Public Health Central Laboratory of Mato Grosso do Sul
PBA	Pseudobulbar affect
RESP-*Microcephalia*	*Registro de Eventos em Saúde Pública* (Public Health Event Registration)
RT-PCR	Reverse transcription polymerase chain reaction
SD	Standard deviation
STORCH	Syphilis, Toxoplasmosis, Rubella, Cytomegalovirus, and Herpes
SINAN	Braziliam National System of Disease Notification
SINASC	Brazilian Live-born Information System
ZIKV	Zika virus
